# Variants That Differentiate Wolf and Dog Populations Are Enriched in Regulatory Elements

**DOI:** 10.1093/gbe/evab076

**Published:** 2021-04-28

**Authors:** Pelin Sahlén, Liu Yanhu, Jinrui Xu, Eniko Kubinyi, Guo-Dong Wang, Peter Savolainen

**Affiliations:** 1 KTH Royal Institute of Technology, School of Chemistry, Biotechnology and Health, Science for Life Laboratory, Stockholm, Sweden; 2 State Key Laboratory of Genetic Resources and Evolution, Kunming Institute of Zoology, Chinese Academy of Sciences, Kunming, China; 3 Program in Computational Biology and Bioinformatics, Yale University, New Haven, Connecticut, USA; 4 Department of Ethology, ELTE Eötvös Loránd University, Budapest, Hungary; 5 Center for Excellence in Animal Evolution and Genetics, Chinese Academy of Sciences, Kunming, China

**Keywords:** domestication, cis-regulatory regions, epigenetics

## Abstract

Research on the genetics of domestication most often focuses on the protein-coding exons. However, exons cover only a minor part (1–2%) of the canine genome, whereas functional mutations may be located also in regions beyond the exome, in regulatory regions. Therefore, a large proportion of phenotypical differences between dogs and wolves may remain genetically unexplained. In this study, we identified variants that have high allelic frequency differences (i.e., highly differentiated variants) between wolves and dogs across the canine genome and investigated the potential functionality. We found that the enrichment of highly differentiated variants was substantially higher in promoters than in exons and that such variants were enriched also in enhancers. Several enriched pathways were identified including oxytocin signaling, carbohydrate digestion and absorption, cancer risk, and facial and body features, many of which reflect phenotypes of potential importance during domestication, including phenotypes of the domestication syndrome. The results highlight the importance of regulatory mutations during dog domestication and motivate the functional annotation of the noncoding part of the canine genome.

**Significance**

Studies for finding genetic variants that mediated the evolution of the dogs from their wolf ancestors have been on the coding part of the canine genome. The role of noncoding variants in cis-regulatory elements is not well studied. We isolated variants that are highly differentiated (HD) between gray wolves and Southeast Asian village dogs and analyzed their distribution in the genome. We found that HD variants are enriched in cis-regulatory elements and this enrichment is larger than that of the protein-coding sequences. We also found that the elements containing HD variants regulate genes that are involved in oxytocin signaling, longevity, and digestion. We hope that our results will motivate a comprehensive annotation of the noncoding canine genome.

## Introduction

The dog was the first domesticated animal and is uniquely integrated into human society. Through domestication, dogs have evolved distinct morphological and behavioral traits which underly their adaptation to the human social environment (Miklósi 2015). Studies of the genetic components behind the evolution of the dog have, so far, focused on the coding part of the genome using variant genotyping and genome sequencing (Vaysse et al. 2011; Plassais et al. 2017, 2019). However, there are many variants in the noncoding part of the wolf and dog genomes, and it is unclear to what extent these variants contribute to phenotypic adaptations. In this study, we set out to answer this question, focusing on enhancers and promoters that are annotated by functional genomic data to increase our detection power.

Promoters and enhancers are the noncoding cis-regulatory elements orchestrating gene expression (Andersson and Sandelin 2020). Several experimental techniques are available for detecting active enhancer regions. Chromatin immunoprecipitation followed by sequencing (ChIP-seq) (Barski et al. 2007) can profile the enhancer signatures, for example, H3K27Ac (Creyghton et al. 2010), H3K4me1 and transcription factor binding sites (Tian et al. 2011). ATAC-seq (Assay for Transposase-Accessible Chromatin using sequencing) is another method to map regulatory elements by locating open chromatin regions (Buenrostro et al. 2013). Although there is a plethora of information regarding the noncoding parts of the genome of humans and some model organisms (Davis et al. 2018), only a few studies are available for mapping enhancer elements in canine genomes. Two major comparative studies (Schmidt et al. 2010; Villar et al. 2015) reported transcription factor binding (*CEBPA* and *HNF4A*) and putative enhancer regions using ChIP-seq in dog livers. A large study, Barkbase, generated open chromatin maps of multiple tissues using ATAC-seq assays (Megquier et al. 2019).

In this study, we assumed that the variants that played significant roles during the evolution of domestic dogs from wolves should show a significant difference in allele frequencies in dogs and wolves. Combining information about the genomic position of regulatory regions and fixation index measure (*F*_ST_) for genome variants from published studies, we identified variants that have high allelic frequency difference between wolves and dogs and that map within enhancers and promoters. We then investigated the potential functionality of the variants that mapped within enhancers and promoters. Our results show that the majority of variants with high *F*_ST_ value are located within promoter and enhancer sequences, many of which are linked to phenotypes of potential importance during domestication, suggesting the importance of changes in gene expression during dog domestication.

## Results

We used publicly available data sets to annotate the enhancers and promoters in the canine genome. Putative enhancer regions covered approximately 5.4% of the genome (supplementary fig. 1 and table 1*a*–*c*, Supplementary Material online). To annotate promoter regions, we used the NCBI RefSeq annotation which includes both curated and predicted genes (supplementary table 1*d*, Supplementary Material online). We analyzed only the promoters of protein coding genes, which resulted in the selection of 24,471 promoters covering 1.7% of the genome (supplementary fig. 1, Supplementary Material online). We also included the exonic sequences in our analyses as a reference, since variants in protein-coding regions were already shown to explain a portion of the phenotypic divergence between dogs and wolves (Axelsson et al. 2013; Cagan and Blass 2016). The exons spanned 1.4% of the genome.

We used the fixation index measure (*F*_ST_) to identify regions with high allelic frequency differences between dogs and wolves, which we call highly differentiated (HD) variants. To capture the general difference between dogs and wolves, and avoid signals from the recent intense selection for extreme morphologic types that formed modern dog breeds, we studied Southeast Asian village dogs, which have a noncontrolled reproduction and nonstandardized morphology and among the highest genetic diversity for dogs around the world, indicating limited population bottlenecks (Boyko et al. 2010; Wang et al. 2016). Therefore, we selected 38 Southeast Asian village dogs and 41 Eurasian and American wolves from a whole genome data set of 722 canids (Plassais et al. 2019), and calculated *F*_ST_ for 19.25 M autosomal SNPs. We focused on the variants with the top 1% *F*_ST_ values for our analyses, the distribution of which is shown in supplementary figure 2*a*, Supplementary Material online (*F*_ST_ values range: [0.54–1]). We then overlapped the positions of these top variants with the promoters and enhancer, finding significantly larger number of SNPs with high *F*_ST_ values in promoters and enhancers than in the genome as a whole (supplementary fig. 2*b*, Supplementary Material online).

### Variants with High Differentiation between Dogs and Wolves Were Enriched in Exon Sequences

**Fig. 1.**
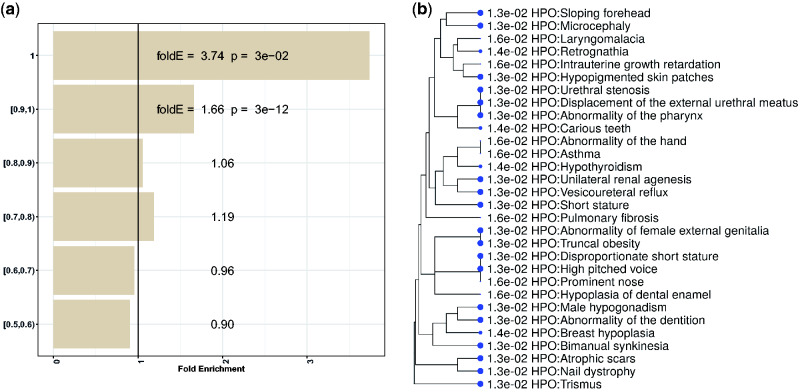
(*a*) Fold enrichment of variants for coding exonic sequences in different *F*_ST_ bins (*y* axis). (*b*) Human phenotypes that are enriched for genes carrying at least one missense exonic variant with an *F*_ST_ value greater than or equal to 0.5. All *P*-values are FDR-corrected and FDR threshold 0.05 is used. The size of the blue dots is proportional to the FDR. The human phenotype ontology database (https://hpo.jax.org/app/) was used for enrichment analyses due to the lack of such functional annotation for the canine genes. The tree is constructed using the distance between two gene sets based on the number of genes in the intersection and the union of two sets. The distance matrix is then used to construct a hierarchical clustering tree based on the number of shared and unique genes between the different sets.

We observed enrichment for variants with high (≥0.9) *F*_ST_ values in exons (fig. 1*a*). We looked at the consequences for all exonic variants with the top 1% *F*_ST_ values which ranges from 0.54 to 1, since *F*_ST_ values as low as 0.3 indicate significant population differentiation (Roux et al. 2016). In order to estimate the functional effect of these variants, we searched for the genes that contained a deleterious HD variant for its function using the SIFT software tool (supplementary table 3, Supplementary Material online). We identified 46 such genes, and although the list was not enriched with significance for any gene ontology category due to the small sample size, there were several marginally enriched phenotypes relating to dental and facial features (fig. 1*b*).

### Regulatory Regions Were Enriched for Variants with High Differentiation between Dogs and Wolves

We then investigated the distribution of the variants in regulatory sequences. We grouped regulatory regions into two groups: Promoters (defined as 1,000 bases upstream and 500 bases downstream of all transcription start sites of all coding transcripts), and enhancers (the nonpromoter regions that were situated within open chromatin regions and/or carried an H3K27Ac mark) (supplementary fig. 1 and methods, Supplementary Material online).

**Fig. 2.**
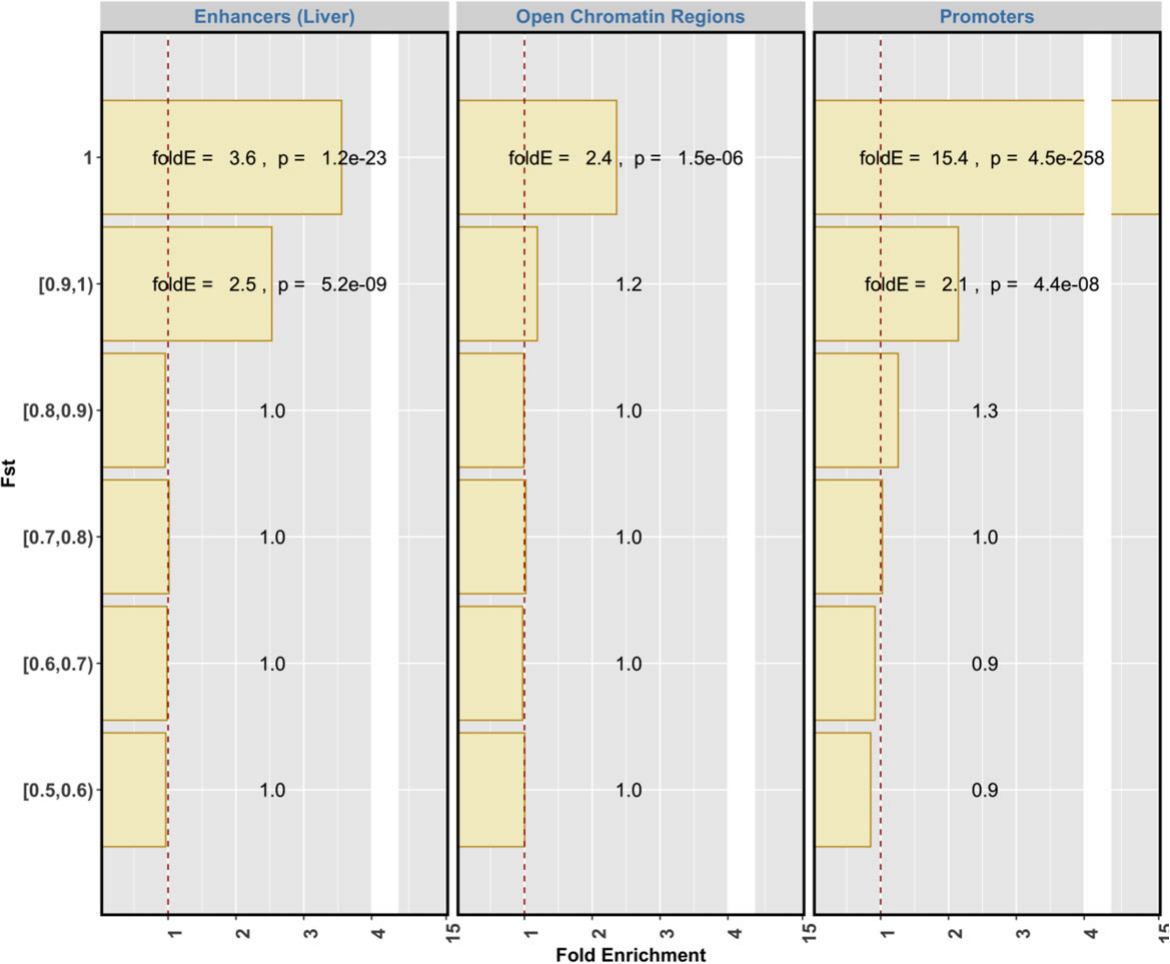
The enrichment of highly differentiated variants between wolves and dogs in regulatory elements; open chromatin regions assayed by ATAC-seq in multiple tissues, enhancers in livers assayed by ChIP-seq against H3K27Ac and promoters of protein-coding genes.

We then overlapped the promoter and enhancer regions with the variants with the top 1% *F*_ST_ values. Out of the variants with *F*_ST_ equal to 1, 20% (169/834) were situated within promoter sequences (15.4-fold enrichment, *P* = 4.5e–258), compared with 9.8% (82/834) for enhancer sequences (3.6-fold enrichment, *P* = 1.2e–23) and 2% (17/834) for exonic sequences (3.74-fold enrichment, *P* = 3e–02) (figs. 1 and 2). Thus, the enrichment in promoters and enhancers was higher than the enrichment in exons and, particularly, the enrichment was substantially higher in promoters than in exons. The variants with *F*_ST_ values greater or equal to 0.9 but less than 1 were also enriched for promoters and enhancers as well as exons, but at similar enrichment levels for all three classes.

### Functional Profile of Enhancers and Promoters Enriched for Variants with High Differentiation between Dogs and Wolves

**Fig. 3.**
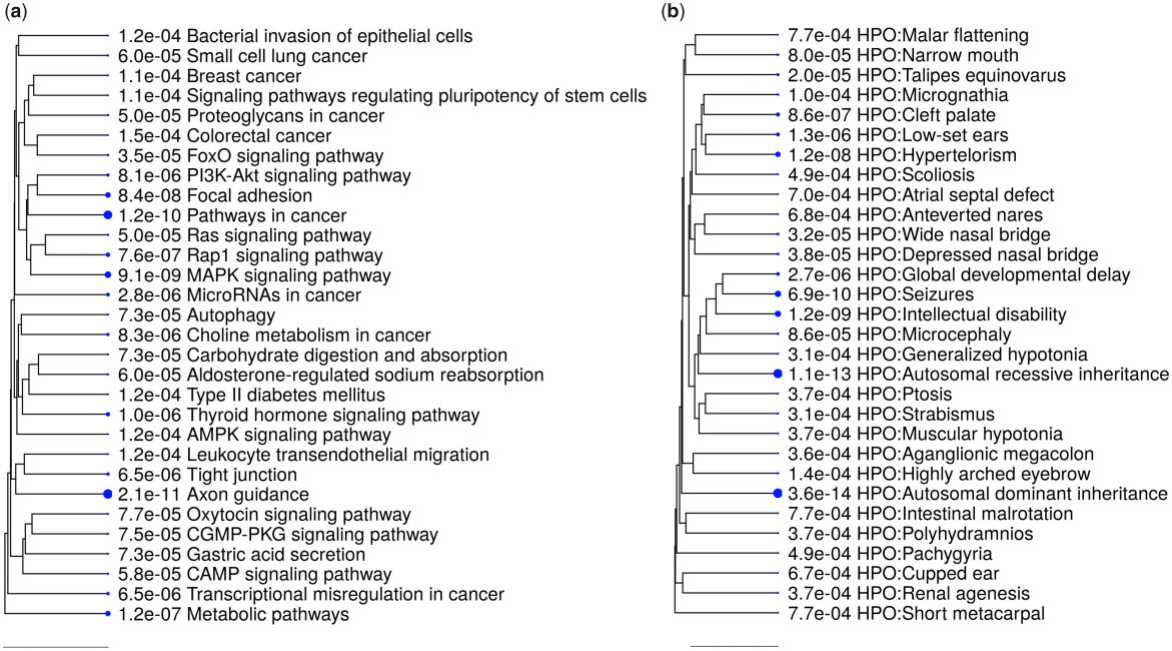
(*a*) The list of KEGG (the database of manually drawn pathway maps) pathways (*b*) human phenotypes (HPO) enriched for promoters regulated by regions with highly differentiated variants between wolves and dogs. FDR threshold of 0.01 is used and only the first 30 terms are shown. The size of the blue dots is proportional to the FDR value. The tree is constructed using the distance between two gene sets based on the number of genes in the intersection and the union of two sets. The distance matrix is then used to construct a hierarchical clustering tree based on the number of shared and unique genes between the different sets.

Our results showed significant enrichment of HD variants within both promoter and enhancer sequences. We, therefore, looked at the functional annotations of genes regulated by the enriched regions. We first performed target gene assignment for the enhancers with HD variants since enhancers are often located far away from the genes they regulate (Akerborg et al. 2019). We used the GREAT software (McLean et al. 2010) which takes gene expression and curated enhancer data sets into account, which should increase the accuracy of the target gene assignment compared with assigning the gene nearest to the enhancer (supplementary material, Supplementary Material online). In our analysis, we only included the enhancers that contained at least one variant in every 500 bases. This resulted in assignment of 2,923 genes to the 5,294 enhancer elements (supplementary fig. 3 and table 4*a* and *b*, Supplementary Material online). Additionally, there were 1,618 genes with promoters containing at least one HD variant which we included. We then performed a functional pathway enrichment analysis using all the genes assigned to enhancers or promoters. Several pathways of potential relevance for dog domestication were enriched, such as oxytocin signaling (Nagasawa et al. 2015) (FDR = 7.7e–5), carbohydrate digestion, absorption (Axelsson et al. 2013) (FDR = 7.3e–5), and longevity regulating pathway (FDR = 8e–4) (fig. 3*a* and supplementary table 4*c*, Supplementary Material online). The term “Pathways in cancer” was one of the most enriched (FDR = 1.2e–10), 30% (160/528) of the genes belonging to this term. Out of the 1,618 genes with promoters containing HD variants, 363 were associated with autosomal dominant diseases (FDR = 3.6e–14) and 534 were associated with autosomal recessive diseases (FDR = 1.1e–13). Many phenotypes related to facial and body features were also enriched, for example, micrognathia, hypertelorism, wide nasal bridge, anteverted nares, and cupped ear (fig. 3*b*).

Interestingly, when we used only the promoters containing the HD variants, only three pathways and four phenotypes were enriched and none of the pathways above were within the enriched terms (supplementary table 5*a*–*c*, Supplementary Material online). However, when only the genes assigned to enhancers were used, almost all of the above terms and phenotypes were enriched but at a lower degree (supplementary table 4*c*, Supplementary Material online). This indicates an important role for enhancers in phenotypic differentiation.

In addition, we selected all promoters that contain HD variants irrespective of whether they overlap with an ATAC-seq or ChIP-seq peak. There were 2,317 such promoters (supplementary table 6*a*, Supplementary Material online). We then investigated if there were particular binding motifs enriched for these promoters, finding that transcription factors, such as CGBP, TET1, DNMT1 were highly enriched (supplementary table 6*b*, Supplementary Material online). These transcription factors are either bound to methylated CpG dinucleotides or are required for DNA cytosine methylation (Tahiliani et al. 2009) and regulate the expression of multiple genes via suppression or activation through DNA methylation.

## Discussion

In this study, we identified functional variants that have substantially different allele frequencies between dogs and wolves and potentially shape the different phenotypes of the dog and wolf populations. Consistent with previous studies, we also found that such variants are enriched in the exons of coding genes. However, for the noncoding genome, promoters and enhancers showed stronger enrichment for the variants, supporting that many adaptive changes are mediated through changes in gene expression levels rather than protein structures (Wray 2007). These results strongly indicate that given the little divergence between wolf and dog proteins, many phenotypic differences can be due to regulatory mutations (King and Wilson 1975; Carroll 2005).

There are mainly two hypotheses that summarize the advantages of using cis-regulatory elements to change phenotypes, compared with using coding genes (Wray 2007). Both hypotheses are based on the flexibility of the cis-regulatory machinery. First, many mutations in cis-regulatory elements can fine-tune the target gene expression. In contrast, only a small portion of mutations are acceptable at protein-coding regions, whereas most mutations likely substantially change the protein stability, and thus drastically reduce the concentration of functional proteins. Consistent with this, the protein coding sequences are under strong purifying selection pressure (Lindblad-Toh et al. 2011; Wang et al. 2013). In addition, since mutations in regulatory elements often act according to additive rather than a recessive model, such mutations can be positively selected immediately (Ruvkun et al. 1991; Consortium 2013; Ponsuksili et al. 2015; Fallahsharoudi et al. 2017; Liu et al. 2020). Second, the mutations in cis-regulatory elements may be less pleiotropic than the mutations in protein-coding regions. For example, one of the cis-regulatory elements of a gene may be used only in a small number of tissues or developmental stages, and thus the mutation associated with the element can fine-tune the target gene expression for particular tissues and stages. By contrast, a nonsynonymous coding mutation permanently impacts the resulting protein (Stern 2000; Wray 2007).

We conducted GO functional analyses of the genes associated with the HD variants. As expected, there were no significantly enriched GO functions in the genes with the variants in their exons, presumably due to the small numbers of such genes. Most of the enriched phenotypes for exonic variants were related to facial and body features. However, we detected more genes whose promoters or enhancers carrying the HD variants. Analyzing these genes confirmed that the enrichment of functions associated with facial and body features was statistically significant, consistent with the domestication syndrome phenomenon (Pendleton et al. 2018). Shorter muzzles, floppy ears, reduced brain size are shared traits among domesticated mammals. Our findings support that these traits are linked, possibly through the mild deficit of the neural crest embryonic development, resulting in “neurocristopathies,” such as micrognathia (reduced jaw size), facial hypoplasia (smaller zygomatic bones), malformed external ear cartilages, and microcephaly (Wilkins et al. 2014).

Moreover, more GO functions, such as digestive functions and cancer-related functions, were also detected. The cancer-related functions are expected to play an important regulatory role in the cell cycle, suggesting substantial changes between dogs and wolves in terms of cell growth, proliferation, and differentiation. These cancer-related functions were enriched in both promoters and enhancers but had higher enrichment levels in enhancers than in promoters. This result supports the notion that enhancers tend to determine cell identities.

We observed that the enrichment for HD variants was higher in promoters than in enhancers, which might be due to the distinct functions between promoters and enhancers. It is widely observed that multiple enhancers are required to interact with one promoter to regulate the expression of its gene in a cell type (Karnuta and Scacheri 2018; Akerborg et al. 2019). This observation suggests that a genetic variant in the promoter may influence the gene expression more directly and effectively, compared with a variant in one of the individual enhancers. Therefore, the adaptive variants in promoters likely have larger effect sizes than those in enhancers, and thus are more likely to become HD variants during domestication.

We also observed that different pathways are associated with enhancers and promoters, respectively, which is likely due to the different regulatory functions of the enhancers and promoters. The enhancers are important to cell-type specific gene expression, and thus determine cell identity, whereas promoters tend to maintain basal gene expression. Due to the cell-type specific functions of the enhancers, the pathways associated with enhancer variants can be different from those associated with promoter variants.

The limitation of this study is that our dog sample (Southeast Asian village dogs) might not well represent the genomic changes that happened during the first step of domestication. Future studies should include a broader geographic sampling of village dogs to verify that the changes we described in this study are generalizable. However, a study (Shannon et al. 2015) including 549 village dogs from 38 countries found strong evidence that dogs were domesticated in Central Asia, in the proximity of Southeast Asia, therefore it is highly likely that our sample faithfully represents the first domesticated dogs.

In summary, this study highlights the importance of regulatory mutations for the study of dog evolution and domestication and will hopefully motivate the annotation of the noncoding canine genomes.

## Supplementary Material

Supplementary data are available at *Genome Biology and Evolution* online.

## Acknowledgements

The computations and data handling were enabled by resources in project [SNIC 2018011] provided by the Swedish National Infrastructure for Computing (SNIC) at UPPMAX, partially funded by the Swedish Research Council through grant agreement no. 2018-05973. This work was supported by a grant from Agria and SKK Forskningsfond.

## Data Availability

All data are incorporated into the article and its supplementary material, Supplementary Material online.

## Supplementary Material

evab076_Supplementary_Data
