# Supplementary material for: Variants That Differentiate Wolf and Dog Populations Are Enriched in Regulatory Elements
Source: Genome Biol Evol. 2021 Apr 28;13(4):evab076. doi: 10.1093/gbe/evab076 (PMC8086526; doi:10.1093/gbe/evab076)
Supplement: evab076_Supplementary_Data [file evab076_supplementary_data.zip › Supplementary Material_revised_v3.docx]

Supplementary Material

# Variants that Differentiate Wolf and Dog Populations are Enriched in Regulatory Elements

Pelin Sahlén^1#^, Liu Yanhu^2^, Jinrui Xu^4^, Eniko Kubinyi^5^, Guo-Dong Wang^2,3^, Peter Savolainen^1^

^1^KTH Royal Institute of Technology, School of Chemistry, Biotechnology and Health, Science for Life Laboratory, Tomtebodavägen 23A, 171 65, Stockholm, Sweden

^2^State Key Laboratory of Genetic Resources and Evolution, Kunming Institute of Zoology, Chinese Academy of Sciences, Kunming, 650223, China

^3^Center for Excellence in Animal Evolution and Genetics, Chinese Academy of Sciences, Kunming, 650223, China

^4^Program in Computational Biology and Bioinformatics, Yale University, New Haven, Connecticut 06520, USA

^5^ELTE Eötvös Loránd University, Department of Ethology, Pázmány Péter sétány 1/C, 1117, Budapest, Hungary

^#^Corresponding author

Pelin Sahlén, [pelin.akan@scilifelab.se](mailto:pelin.akan@scilifelab.se)

Table of Contents

[Variants that Differentiate Wolf and Dog Populations are Enriched in Regulatory Elements 1](#_Toc64631202)

[Methods 3](#_Toc64631203)

[Subjects 3](#_Toc64631204)

[Variant Dataset and Fixation index measure (F_ST_) calculation 3](#_Toc64631205)

[Feature datasets 3](#_Toc64631206)

[Exonic variants 4](#_Toc64631207)

[Generation of Random Datasets 4](#_Toc64631208)

[Calculation of Fold Enrichment 4](#_Toc64631209)

[Target Gene Assignment 4](#_Toc64631210)

[Supplementary Figures 6](#_Toc64631211)

[Supplementary Figure 1 6](#_Toc64631212)

[Supplementary Figure 2 7](#_Toc64631213)

[Supplementary Tables 7](#_Toc64631214)

[Supplementary Table 1 7](#_Toc64631215)

[Supplementary Table 2 7](#_Toc64631216)

[Supplementary Table 3 7](#_Toc64631217)

[Supplementary Table 4 8](#_Toc64631218)

[Supplementary Table 5 8](#_Toc64631219)

[Supplementary Table 6 8](#_Toc64631220)

[References 8](#_Toc64631221)

# Methods

## Subjects

41 wolves from Eurasia and America and 38 Southeast Asian village dogs were chosen from a dataset of 722 canids. The Southern Chinese village dogs were differentiated from Northern Chinese dogs based on the sample identifications in NCBI.

## Variant Dataset and Fixation index measure (F_ST_) calculation

The raw SNP files of 722 individuals were downloaded from NCBI (<https://www.ncbi.nlm.nih.gov/bioproject/PRJNA448733>, (Plassais et al. 2019). Five grey wolves were removed due to admixture with coyotes (Sinding et al. 2018). SNPs in autosomes marked by PASS were used for analysis. F_ST_ for sites was calculated between dogs and wolves by VCFtools (version v0.1.13, (Danecek et al. 2011). Top1% Fst sites were carried out for annotation.

## Feature datasets

The enhancer dataset was generated by merging three public datasets. We downloaded the ATAC-seq peaks from <https://data.broadinstitute.org/barkbase/ATAC-Seq_peak_files/> publicly available from the BarkBase (Megquier et al. 2019) project. We only took tissues that had at least two replicates and took only those peaks that were replicated at least two times. We also removed tissues that had very little to no overlap with the variants (less than 10 overlaps). The following tissues were then left for the analysis: bone marrow, left atrium, liver, lymph node, pancreas, pituitary gland, right ventricle, parotid salivary gland, spleen and stomach (Supplementary Table 1a). The first dataset was taken from Villar et al. (2015), and downloaded from the public repository E-MTAB-2633. Only the replicated H3K27Ac peaks were taken for the study to not include possibly false positive regions due to technical noise in the experiments (Supplementary Table 1b). We also downloaded raw fasta files from the public repository E-TABM-722 for binding sites liver transcription factor CEBPA and HNF4A (Supplementary Table 1c). The fasta files were mapped to canFam3.1 assembly with Bowtie 2 (version 2.3.5.1, (Langmead and Salzberg 2012) using default settings. The duplicate reads were removed using PicardTools (<https://github.com/broadinstitute/picard>) and peaks were called using MACS2 package (18798982) using a q value cutoff 0.01. We removed any peak that overlapped with any promoter sequence (described below) from the enhancer dataset.

The promoter dataset was generated using NCBI RefSeq annotation (NCBI RefSeqAll), available for CanFam3 assembly that contains both curated and predicted genes (Supplementary Table 1d). The exons were also taken from the same RefSeq annotation.

## Exonic variants

We used Ensembl Variant Effect Predictor (VEP, (McLaren et al. 2016) to predict the consequence of exonic variants. We evaluated the severity of the mutation and chose only genes with MODERATE and HIGH effect and/or containing “deleterious” mutations according to SIFT (Vaser et al. 2016) score for the enrichment analyses.

## Generation of Random Datasets

We used the *bedtools shuffle* function to generate random datasets for enhancer, promoter and exon datasets. All regions were size and length matched. Any region from either of the actual enhancer and promoter datasets was excluded from the random datasets.

## Calculation of Fold Enrichment

We calculated the overlap of each genomic feature dataset to each binned variant set using the *bedtools intersect* function. We then generated ten random genomic feature datasets matched by size, and also overlapped those with each binned variant set. We then calculated a z score by comparing the real overlap value (RealOverlap) to the mean and standard deviation of overlap values of ten random datasets (mRandomOverlap), calculated a p value assuming a normal distribution. We also generated a random set of variants (by randomly selecting 10% of the total variant set) and overlapped them with each genomic feature set to determine the background enrichment of the variants (BackgroundOverlap) in any genomic feature. Fold enrichment was calculated by:

FoldE = ($\frac{RealOverlap}{mean(RandomOverlap)})/BackgroundOverlap$

## Target Gene Assignment

We used two different approaches for target gene assignment to enhancer regions. First, we used *bedtools closest* function to assign the nearest gene to the enhancer. However, since only one-third of the time an enhancer regulates its nearest gene (Akerborg et al. 2019), we also used the GREAT software (McLean et al. 2010) that assigns genes based on tissue-specific gene expression and enhancer information. Since GREAT is only available for humans and a few model organisms, we converted variant coordinates from canFam 3.1 to hg38 assembly. We took 50 bases upstream and downstream of the variant and required at least 50% base identity for conversion using the UCSC *LiftOver* tool (<https://genome.ucsc.edu/cgi-bin/hgLiftOver>). When we performed enrichment analyses using both the nearest gene approach and the GREAT software, most of the enriched pathways were the same, except that GREAT software detected stronger enrichments, suggesting more accurate assignment (data not shown). GREAT software combines many different datasets such as gene expression, gene ontology, phenotype and disease associations and enhancer annotation datasets (ChIP-seq experiments for different histone modifications and transcriptions factors in different tissues and developmental context) to assign each gene to a regulatory domain i.e. a putative enhancer region. Then it uses this association information to find the best mapping for the regions given by the user. Since it uses contextual information (such as gene expression, annotation, etc.) to map the genes to the regulatory domains, it outperforms the nearest gene mapping where the nearest gene is mapped to the region of interest independent of gene expression or annotation profile.

We used ShinyGO (Ge et al. 2020) to perform all enrichment analyses and used FDR threshold of 0.01. For the TF enrichment analyses of promoters, we used 300 bases upstream of the promoter for the motif search.

# Supplementary Figures

## Supplementary Figure 1

Supplementary Figure 1. Overlap status of promoter and enhancer datasets used in the study. Numbers in parentheses denote the number of bases covered by each of the corresponding set.

## Supplementary Figure 2


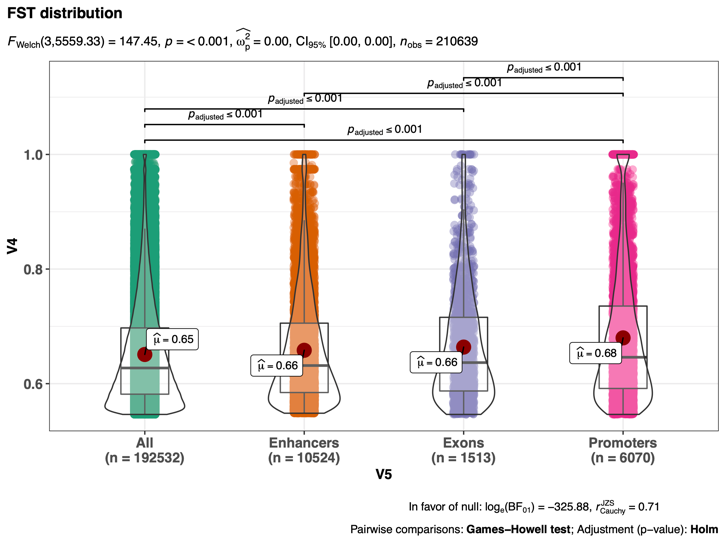


a

b

**Supplementary Figure 2.** a) The number and percentage of variants with different F_ST_ values within the top 1% Fst variants. F_ST_ values are binned into six intervals with a bin size of 0.1. b) The enrichment of SNPs with top 1% Fst values in promoter, enhancer and exonic regions. “ggstatsplot” (https://github.com/IndrajeetPatil/ggstatsplot) R package was used for the comparisons.

# Supplementary Tables

## Supplementary Table 1

Supplementary Table 1a: All ATAC-seq peaks used in this study. The peaks are downloaded from http://www.barkbase.org/

Supplementary Table 1b: All Histone H3K27Ac peaks used in this study, downloaded from E-MTAB-2633 repository

Supplementary Table 1d: CEBP and HNF4A binding sites, raw files are downloaded from E-TABM-722 repository

Supplementary Table 1d: The RefSeq All gene annotations used for this study, as downloaded from the UCSC Genome Table Browser

## Supplementary Table 2

The list of all variants used in this study. Please see methods for more details

## Supplementary Table 3

The consequences of top 1% F_ST_ exonic variants, ENSEMBL Variant Effect Predictor (VEP, <http://www.ensembl.org/info/docs/tools/vep/index.html>) and SIFT (<https://sift.bii.a-star.edu.sg/>) is used to predict the functional outcome of each variant with top 1% F_ST_ within exons

## Supplementary Table 4

Supplementary Table 4a: All enhancer regions that contain at least one variant with a top 1% F_ST_ value. Enhancers are defined as regions that overlap with at least one region in Supplementary Table 1-c

Supplementary Table 4b: Target genes assigned to the enhancers using GREAT software using hg38 coordinates in Supplementary Table 4a

Supplementary Table 4c: Enriched KEGG pathways using only the genes in Supplementary Table 4b

## Supplementary Table 5

Supplementary Table 5a: The promoters (listed in Supplementary Table 1d) that overlap with at least one ATAC-seq, Liver ChIP-seq peak and contain at least one variant with top 1% F_ST_ value

Supplementary Table 5b: The target gene assignment by GREAT software. This was to improve the mapping and remove artificial mappings due to possible poor gene annotation in canFam3 assembly

Supplementary Table 5c: Enriched KEGG pathways and human phenotypes using only the genes in Supplementary Table 5b

## Supplementary Table 6

Supplementary Table 6a: All the promoters that contain at least one variant with a top 1% F_ST_ value

Supplementary Table 6b: Transcription factor (TF) binding motifs that are enriched for promoters containing at least one dog-wolf-specific variant. ShinyGO application is used for the analysis

# References

Akerborg O, Spalinskas R, Pradhananga S, Anil A, Hojer P, Poujade FA, Folkersen L, Eriksson PP, Sahlen P. 2019. High-Resolution Regulatory Maps Connect Vascular Risk Variants to Disease-Related Pathways. Circ Genom Precis Med 12(3):e002353.

Danecek P, Auton A, Abecasis G, Albers CA, Banks E, DePristo MA, Handsaker RE, Lunter G, Marth GT, Sherry ST. 2011. The variant call format and VCFtools. Bioinformatics 27(15):2156-2158.

Ge SX, Jung D, Yao R. 2020. ShinyGO: a graphical gene-set enrichment tool for animals and plants. Bioinformatics 36(8):2628-2629.

Langmead B, Salzberg SL. 2012. Fast gapped-read alignment with Bowtie 2. Nat Methods 9(4):357-9.

McLaren W, Gil L, Hunt SE, Riat HS, Ritchie GR, Thormann A, Flicek P, Cunningham F. 2016. The Ensembl Variant Effect Predictor. Genome Biol 17(1):122.

McLean CY, Bristor D, Hiller M, Clarke SL, Schaar BT, Lowe CB, Wenger AM, Bejerano G. 2010. GREAT improves functional interpretation of cis-regulatory regions. Nat Biotechnol 28(5):495-501.

Megquier K, Genereux DP, Hekman J, Swofford R, Turner-Maier J, Johnson J, Alonso J, Li X, Morrill K, Anguish LJ et al. . 2019. BarkBase: Epigenomic Annotation of Canine Genomes. Genes (Basel) 10(6).

Plassais J, Kim J, Davis BW, Karyadi DM, Hogan AN, Harris AC, Decker B, Parker HG, Ostrander EA. 2019. Whole genome sequencing of canids reveals genomic regions under selection and variants influencing morphology. Nature Communications 10(1):1489.

Sinding MS, Gopalakrishan S, Vieira FG, Samaniego Castruita JA, Raundrup K, Heide Jorgensen MP, Meldgaard M, Petersen B, Sicheritz-Ponten T, Mikkelsen JB et al. . 2018. Population genomics of grey wolves and wolf-like canids in North America. PLoS Genet 14(11):e1007745.

Vaser R, Adusumalli S, Leng SN, Sikic M, Ng PC. 2016. SIFT missense predictions for genomes. Nat Protoc 11(1):1-9.

Villar D, Berthelot C, Aldridge S, Rayner TF, Lukk M, Pignatelli M, Park TJ, Deaville R, Erichsen JT, Jasinska AJ et al. . 2015. Enhancer evolution across 20 mammalian species. Cell 160(3):554-66.
